# Advances in Research on the circRNA-miRNA-mRNA Network in Coronary Heart Disease Treated with Traditional Chinese Medicine

**DOI:** 10.1155/2020/8048691

**Published:** 2020-02-17

**Authors:** Fei Lin, Heng-Wen Chen, Guo-An Zhao, Yan Li, Xuan-Hui He, Wan-Qian Liang, Zhuo-Lin Shi, Si-Yu Sun, Pan-Pan Tian, Ming-Yan Huang, Chao Liu

**Affiliations:** ^1^Department of Cardiology, The First Affiliated Hospital of Xinxiang Medical University, Xinxiang 453100, Henan, China; ^2^Department of Cardiology, Guang'anmen Hospital, China Academy of Chinese Medical Sciences, Beijing 100053, China

## Abstract

There has been an increase in morbidity and mortality related to coronary heart disease (CHD) in China in recent years. Numerous clinical experiences and studies have shown that traditional Chinese medicine (TCM) plays an important role in the prevention, treatment, and prognosis of CHD. However, the mechanism of TCM in the treatment of CHD has not yet been elucidated. The circRNA-miRNA-mRNA network consists of miRNA that is competitively bound by circRNA, and miRNA regulates the transcription level of mRNA. Through literature review, we found that the circRNA-miRNA-mRNA network acts to contribute to certain effects to CHD such as myocardial hypertrophy, myocardial fibrosis, and heart failure. TCM contains constituents that act against CHD by antiatherosclerosis and apoptosis inhibition action, cardiac and cardiomyocyte protection, and these components also promote cell growth and protection of the vascular system by regulating miRNAs. Therefore, we consider that the circRNA-miRNA-mRNA network may be a new regulatory mechanism for the effective treatment of CHD by TCM.

## 1. Introduction

The morbidity and mortality caused by coronary heart disease (CHD) are steadily increasing in China. According to a 2018 report on cardiovascular disease in China, in 2013, 11,396,104 people died from CHD, with CHD mortality in 2016 of 113.46/100 thousand for urban residents and 118.78/100 thousand for rural residents. Therefore, it is urgent to find effective methods for the prevention and treatment of CHD events [1].

The etiology of CHD is cavity vascular stenosis, coronary insufficiency, acute or transient ischemia, and hypoxia of myocardial cells caused by coronary atherosclerosis (AS). CHD belongs to the *XiongBi* or *heartache* category in traditional Chinese medicine (TCM). With a history of thousands of years, TCM is unique in theory and rich in clinical practice. Syndrome differentiation and treatment and holistic concept are two basic characteristics of TCM. A syndrome reflects the pathological essence of a certain stage in the development of disease. Phlegm and blood syndrome (PBSS) is one of the common syndrome types in CHD, and its formation and development are closely related to blood stasis, obesity, hyperlipidemia, hyperglycemia, body deficiency, old age, and environment. For PBSS, a satisfactory clinical outcome is achieved by promoting blood circulation and dispersing phlegm. There are three types of Chinese medicine used for the prevention and treatment of CHD, namely, a single Chinese medicine (e.g., redroot salvia, Rhodiola, and pseudoginseng), effective ingredients (e.g., tanshinone IIA, salvianolic acid, and ligustrazine), and prescriptions (e.g., Xuefu-Zhuyu decoction, *Chaihu-Shugan* powder, *Gualou-Xiebai-Banxia* decoction, and Ditan decoction). However, the mechanisms of these Chinese herbal medicines (CHMs) have not been fully elucidated.

Circular RNAs (circRNA) and micro-RNAs (miRNA) are noncoding RNAs and have been confirmed to contribute to the pathogenesis and progression of CHD [2, 3]. CircRNA and miRNA interact with each other and regulate the mRNA expression of critical genes in CHD genesis and progression. Therefore, we speculated that there is a connection between the circRNA-miRNA-mRNA network and TCM and gathered supporting data through reviewing and summarizing the literature.

## 2. The Therapeutic Effect of TCM in CHD

TCM aims to treat CHD based on holistic regulation and syndrome differentiation and treatment. According to the model theory of gene constitution syndrome pattern, the occurrence and development of TCM syndromes are mainly based on the genomics of gene expression, which is a powerful approach to gain insight into the prevention and treatment of diseases [4].

In TCM, CHD is also described as *XiongBi*, which belongs to the category of chest pain, thoracic obstruction, heartache, or angina pectoris as a consequence of cold invasion, improper diet, mood disorder, fatigue, bodily internal injury, or body deficiency due to old age. CHD entails mainly obstruction of the heart channel with injury to other organs including the lungs, liver, spleen, and kidneys [5]. The syndromes of CHD in Chinese clinical medicine manifest as intermingled deficiency in origin (mostly *Qi*, blood, but also *Qi-Yin* and *Yang*) and excess in superficiality (mainly blood stasis and phlegm turbidity, but also cold coagulation and *Qi* stagnation) [6]. *Qi* in the TCM is a kind of substance with strong vitality and constant movement and extremely subtle, which is the unity of life substance and physiological function. *Yin* and *Yang* represent two opposite aspects of every object and their implicit conflict and interdependence. Generally, anything that is moving, ascending, bright, progressing, and hyperactive, including functional disease of the body, pertains to yang. The characteristics of stillness, descending, darkness, degeneration, and hypoactivity, including organic disease, pertain to *Yin*.

Traditional Chinese syndromes in *XiongBi* involve (a) heart-blood stasis syndrome (*Xin Xue Yu Zu*), (b) cold coagulation in heart-vessel syndrome (*Han Ning Xin Mai*), (c) *Qi* stagnation blood stasis syndrome (*Qi Zhi Xin Xiong*), (d) phlegm stasis in heart-vessel syndrome (*Tan Zhuo Bi Zu*), (e) deficiency of both *Qi* and *Yin* syndrome (*Qi Yin Liang Xu*), (f) *Yin* deficiency of heart and kidney syndrome (*Xin Shen Yin Xu*), and (g) *Yang* deficiency of heart and kidney syndrome (*Xin Shen Yang Xu*). These syndrome types are shown in Table 1 [6].

Based on the principle of holistic regulation and syndrome differentiation and treatment, CHMs have been widely used in disease prevention and treatment in China and have been proven to have great clinical effectiveness on cardiovascular system disease. A previous study that summarized and introduced the application of plant products in CHD, AS, dyslipidemia, and hypertension also sorted formulae and herbs according to specific cardiovascular diseases [7]. The plant products applied in AS and dyslipidemia were *Allium ﬁstulosum* L. (Amaryllidaceae), *Allium sativum* L. (Amaryllidaceae), *Astragalus propinquus* (Fabaceae), *Coptis chinensis* Franch. (Ranunculaceae), *Crataegus* spp. (Rosaceae), *Epimedium brevicornum* Maxim (Berberidaceae), *Fallopia multiﬂora* Thunb. (Polygonaceae), *Fermentum rubrum* (Aspergillaceae), *Olea europaea* L. (Oleaceae), *Panax ginseng* C.A. Mey (Araliaceae), *Pueraria lobata (*Willd*) ohwi* (Leguminosae), *Reynoutria japonica* Houtt (Polygonaceae), *Rheum palmatum* Linn. (Polygonaceae), *Salvia miltiorrhiza* Bunge (Lamiaceae), *Scutellaria baicalensis* Georgi (Lamiaceae), *Senna obtusifolia* Linn. (Fabaceae), *Cassia tora* Linn. (Leguminosae), *Ligusticum chuanxiong* Hort. (Umbelliferae), *Rhodiola crenulata* H. Ohb (Crassulaceae), and *Curcuma longa* L. (Gingeraceae) [7–10].

Over the last decades, some TCM decoctions have been confirmed to be effective in the treatment of CHD. For instance, combination therapy with *Xuefu Zhuyu* decoction and traditional antianginal medications (TAMs) was more effective in treating angina pectoris than TAMs alone. Evidence has indicated that *Xuefu Zhuyu* decoction combined with TAMs was more effective in improving RAS (RR = 1.29; 95% CI = (1.20, 1.38)), ECG (RR = 1.37; 95% CI = (1.22, 1.54)), and blood HDL-C level (MD = 0.29 mmol/L; 95% CI = (0.23, 0.35)) compared with TAMs alone from a meta-analysis and systematic review of 14 randomized controlled trials [11]. There was benefit in the use of *Tongxinluo* (TXL) capsule for secondary prevention after acute myocardial infarction (AMI). A systematic review and meta-analysis of randomized controlled trials indicated that TXL improves cardiac function; regulates blood lipid total cholesterol (TC), triglycerides (TG), and low-density lipoprotein-C (LDL-C); and decreases the level of hs-C-reactive protein (hs-CRP) [12]. A meta-analysis indicated that Huoxue Huayu therapy (HXHY) is an effective and safe therapy for patients after percutaneous coronary intervention (PCI). HXHY had a greater beneficial effect on reducing the in-stent restenosis (ISR) rate and the degree of restenosis, improving Seattle Angina Questionnaire (SAQ) scores and increasing the revascularization rate compared with placebo. However, the rate of death and myocardial infarction (MI) of patients treated with HXHY was no different from that of those treated with placebo (*P* > 0.05) [13].

According to new studies, the pharmacological mechanisms of CHM are relevant to the effects on vascular smooth muscle cells (VSMCs), endothelial cells, cardiomyocytes, macrophages, and monocytes. Details are shown in Table 2 [7].

## 3. Regulation of miRNAs in CHD

With the improvement in modern living standards, phlegm and blood stasis commonly occur and then aggravate each other during CHD progression [14, 15]. Phlegm is a pathological substance caused by the disturbance of body fluid. Blood stasis is a form of pathology caused by the disturbance of blood circulation. Research on CHD with PBSS has mainly focused on lipid metabolism, inflammatory factors, hemorheology, coagulation function, endothelial cell injury, endoplasmic reticulum autophagy, insulin resistance, genomics, proteomics, and metabolomics [16].

MiRNAs are nuclear genome-encoded single-stranded small (18−23 bases) RNAs that are highly expressed in heart tissue, and they play central roles in the miRNA-miRNA functional synergistic network [17]. The specificity and timing of miRNA expression are very similar to the dynamic spatiotemporal characteristics of TCM syndromes. The expression of miR-146b-5p, miR-199a-3p, and miR-199a-5p is upregulated, CALR and TP53 are downregulated in blood stasis syndrome, and miR-363-5p and miR-668 are downregulated, and RIPK2 and STK4 are upregulated in phlegm-turbidity syndrome. In PBSS, the expression of miR-1207-5p, miR-1321, miR-320d, miR-765, and other miRNAs is upregulated, while the expression of miR-1181, miR-1225-3p, miR-1248, miR-668, miR-1223, miR-1281, miR-1538, miR-181d, miR-342-3p, miR-483-3p, miR-491-3p, miR-494, miR-532-3p, miR-668, and miR-98 is downregulated [18–20]. Peroxisome proliferator-activated receptor-*α* (PPAR-*α*) is an important factor affecting PBSS that can be regulated by miR-21 [21]. MiR-4433-3p and miR-363-5p are downregulated in patients with *Qi* deficiency, phlegm, and blood stasis syndrome. In patients with *Qi* deficiency and blood stasis syndrome, miR-17-5P, miR-320a, and miR-320c are downregulated [22].

The target genes of miRNA related to CHD with PBSS may regulate cell signaling pathways through the transforming growth factor-*β* (TGF-*β*) signaling pathway. MiRNAs such as miR-107, miR-146b-5p, miR-199a-5p, and miR-661 are upregulated, while miR-1321 is downregulated after *HuoxueHuatan Anshen* formula intervention in angina pectoris CHD with PBSS [23]. In addition, TGF-*β*1 is the initiating factor for synthesizing and depositing collagen fibers and other extracellular active components. After AMI, miR-21 induces myocardial fibrosis through the TGF-0205/Smad7 pathway [24], and circRNA-010567 promotes myocardial fibrosis by inhibiting miR-141 via targeting TGF-*β*1 [25]. In addition, abnormal proliferation or migration of VSMCs can lead to vessel lesions, resulting in AS and in stent-restenosis. The expression of miR-378a-5p was increased in the group with stent restenosis compared with healthy people, and miR-378a-5p overexpression promoted proliferation and migration in VSMCs by targeting CDK1 [26]. The changes in VSMC phenotype and vascular calcification are major characteristics of AS. VSMCs express miRNAs, and there is a causal link between miRNAs such miR-21, miR-125b, miR-133a, miR-136, miR-143/145, miR-1, and miR-206 and the pathogenesis of vascular disorders in cardiovascular disease with diabetes mellitus [27]. It is important that miRNAs such as miRNA-143/-145, miR-133, miR-125a-5p, miR-23b, miR-638, miR-663, miR-21, miR-100, miR-143, and miR-145 can regulate VMSC function, and miR-126-3p, miR-126-5p, miR-92a, miR-221, and miR-222 can also regulate endothelial cell function [28, 29].

The various aspects of treatment for CHD with PBSS are also closely related to miRNAs. Treatment of spleen phlegm-turbidity and blood stasis syndrome of CHD may be accomplished via multiple targets, including miRNA [30]. Studies have detected 12 differentially expressed miRNAs in dyslipidemia patients with spleen deficiency syndrome, and the results showed 10 downregulated (miR-124-3p, miR-9-5p, miR-133b, miR-1, miR-136-5p, miR-144-3p, miR-133a, miR-149-5p, miR-200a-3p, and miR-219-5p) and 2 upregulated (miR-193a-5p, miR-665) miRNAs that were involved in fatty acid metabolism, phagocytosis, and proliferation- and apoptosis-related signal pathways [31]. In addition, miRNAs can discriminate the severity of blood stasis syndrome, allowing prognostication for patients with acute coronary syndrome. For example, miR-208a-3p expression can be used to distinguish acute coronary syndrome patients with blood stasis syndrome from healthy volunteers. MiR-222-3p and miR-198 may help to grade the severity of blood stasis [32]. Additionally, it was reported that TCM remedies contain active components that can protect cardiomyocytes, protect against AS, inhibit apoptosis, promote cell growth, and protect the vascular system by regulating miRNAs. Details are shown in Tables 3 and 4.

## 4. CircRNA-miRNA-mRNA in CHD

CircRNA is an endogenous noncoding RNA. It has no 5′-end cap or 3′-end poly (A) tail as a covalently closed loop from precursor mRNA back-splicing by the spliceosome machinery. Some circRNAs have been shown to act as miRNA sponges, to interact with RNA-binding proteins, to regulate transcription, or to be translated into proteins. CircRNAs have been widely explored in antiaging strategies, diabetes, cardiovascular diseases, and malignant tumors [44–50]. In various cardiovascular tissues and organs, circRNA participates in physiological and pathological processes such as myocardial repair regulation and fibrosis of vascular smooth muscle cells and myocardium, without being affected by the expression of maternal genes. Thus, circRNAs affect the occurrence of cardiovascular diseases such as heart failure and hypertension. CircRNAs and miRNAs interact with each other in CHD genesis and progression. The circRNA-miRNA network may regulate mRNA participating in the development of CHD through competitive endogenous RNA (ceRNA). A new regulation model of gene expression and a complex network system has been developed that involves ceRNA, in which coding RNA and noncoding RNA interact with each other. This regulatory network is constructed to affect the mutual regulation, restriction, and interaction among lncRNA, circRNA, miRNA, and mRNA, thus affecting the target genes participating in CHD [51].

The circRNAs that have been reported to be differentially expressed in *Qi* stagnation and blood stasis syndrome include circRNA_0_9849, circRNA_1_1523, circRNA_1_8046, and circRNA_2_4450, with circRNA 11523 being related to hsa-circ-0005860 in circBase [52]. Through the KEGG pathway enrichment analysis of differential circRNA in *Qi* stagnation and blood stasis syndrome, five enrichment pathways were found, namely, path:hsa05144 (malaria), path:hsa04914 (progesterone-mediated oocyte maturation), path:hsa04650 (natural killer cell-mediated cytotoxicity), path:hsa04110 (cell cycle), and path:hsa05203 (viral carcinogenesis). These circRNAs also participate in the regulation of natural killer cell-mediated cytotoxicity and cell cycle. Previous studies showed that a total of 1670 circRNAs and 13 miRNAs were differentially expressed in AMI, and 110 circRNAs and 11 miRNAs were differentially expressed in CHD. Gene ontology (GO) and pathway analyses for differentially expressed circRNAs showed that many pathways, diseases, and functions participate in the development of AMI [53]. One study also showed that there were nine circRNAs, namely, hsa_circ_0089378, hsa_circ_0083357, hsa_circ_0082824, hsa_circ_0068942, hsa_circ_0057576, hsa_circ_0054537, hsa_circ_0051172, hsa_circ_0032970, and hsa_circ_0006323, that could negatively regulate miR-130A-3p, resulting in the upregulation of transient receptor potential cation channel subfamily M member 3 (TRPM3) in patients with CHD, with nine circRNAs promoting TRPM3 expression by inhibiting hsa-miR-130a-3p in CHD patients [54]. The effects of the circRNA-microNA-mRNA network on CHD include myocardial hypertrophy, myocardial fibrosis, and heart failure, which are shown in Table 5.

Adrb1: adrenoceptor beta 1; Adcy6, adenylate cyclase 6; ARC: activity‐regulated cytoskeletal protein; CDR1as: Cerebellar degeneration-related protein 1 antisense ciRS-7; CDIP1: cell death-inducing protein 1; circANRIL: Circular antisense non-coding RNA in the INK4 locus; COL1A1: type I collagen; COL3A1: type III collagen; EGR1: early growth response protein 1; CTGF: connective tissue growth factor; FOXO1: Forkhead Box Protein O1; Gsk3*β*: glycogen synthase kinase 3*β*; IER2: immediate early response protein 2; MFACR: mitochondrial fission and apoptosis-related circRNA; MTP18: mitochondrial protein, 18 kDa; NCX1 sodium/calcium exchanger 1 (ncx1) gene; NR4A1: nuclear receptor subfamily 4 group A member 1; NRG-1: Neuregulin-1; PARP: Poly (ADP-ribose) polymerase; PES1: pescadillo homologue 1; *α*-SMA: *α*-smooth muscle actin; SP1: transcription factor Sp1; SRF: serum response factor; STIM1: stromal interaction molecule 1; TGF-*β*1: transforming growth factor-*β*1.

CircRNAs with a covalently closed continuous loop are an abundant class of endogenous RNAs that are formed during the maturation of precursor mRNA, and they have been widely researched with respect to cardiovascular diseases. When CircANRIL binds to pescadillo homologue 1 (PES1), it impairs exonuclease-mediated pre-rRNA processing and ribosome biogenesis in vascular smooth muscle cells and macrophages and induces nucleolar stress and p53 activation, resulting in the induction of apoptosis and inhibition of proliferation for conferring atheroprotection [55]. Cdr1as also functioned as a powerful miR-7a sponge in myocardial cells, involving the function of miR-7a targets PARP and SP1 in MI injury [56]. CircRNA HRCR can sequester and inhibit miR-223 activity and was then used in the treatment of cardiac hypertrophy and heart failure through increasing the ARC expression [57]. MFACR regulates mitochondrial dynamics and apoptosis through miR-652-3p and MTP18 [58]. CircRNA_010567 promotes myocardial fibrosis via suppressing miR-141 by targeting TGF-*β*1 [25]. CircNCX1 promotes cardiomyocyte apoptosis via the miR-133a-3p/CDIP1 [59]. Circ-SATB2 can regulate vascular smooth muscle cell phenotypic differentiation, proliferation, apoptosis, and migration by promoting the expression of STIM1 (a target gene of miR-939) [60]. CircCHFR facilitates the proliferation and migration of vascular smooth muscle via the miR-370/FOXO1/cyclin D1 pathway [61]. CircSlc8a1 can function as an endogenous sponge for miR-133a in cardiomyocytes, and it can regulate the serum response factor, connective tissue growth factor, adrenoceptor beta 1, and adenylate cyclase 6 through directly intervening the knockdown and overexpression in heart failure [62]. CircNfix can suppress Ybx1 ubiquitin-dependent degradation and increase miR-214 activity. It also can promote cardiac regenerative repair and functional recovery after MI [63]. Overexpression of circHIPK3 reverses miR-29b-3p-induced inhibition of cardiac fibrosis proliferation and migration, and then alters the expression levels of miR-29b-3p-targeting genes (a-SMA, COL1A1, COL3A1) in vitro [64].

## 5. Potential circRNA-miRNA-mRNA Network in CHD Treated with TCM

**Figure 1:**
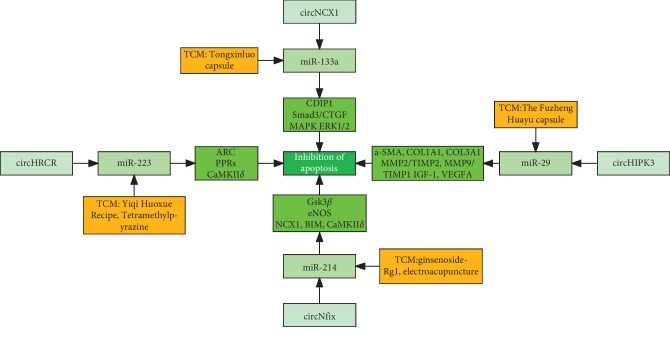
The potential role of the circRNA-miRNA-mRNA regulatory network in the cardiovascular diseases treated with TCM CDIP1: cell death-inducing protein 1; Smad3: recombinant human mothers against decapentaplegic homolog 3; CTGF: connective tissue growth factor; MAPK: mitogen-activated protein kinase; ERK1/2: extracellular signal regulated kinases 1 and 2; ARC: recombinant activity regulated cytoskeleton associated protein; PPRs: pentatricopeptide repeat proteins; CaMKIIδ: calmodulin-dependent protein kinase II*δ*; GSK3*β*: glycogen synthase kinase-3*β*; eNOS: endothelial nitric oxide synthase; NCX1: sodium/calcium exchanger 1; BIM: BCL2-like 11; a-SMA: anti-*α* smooth muscle actin; COL1A1: recombinant collagen type I alpha 1; COL3A1: recombinant collagen type III alpha 1; MMP: mitochondrial membrane potential; TIMP: tissue inhibitor of metalloproteinase; IGF-1: insulin-like growth factor-1; and VEGFA: vascular endothelial cells growth factor A.

TCM may play a positive role in the treatment of cardiovascular diseases through the circRNA/miRNA/mRNA regulatory network (Figure 1). In myocardial ischemia-reperfusion injury, increasing amounts of circNCX1 competitively bind to the miRNA-133a family, decreasing miRNA-133a-3p levels and increasing the activity of CDIP1, leading to cardiomyocyte deterioration [59]. Tongxinluo can increase the expression of the miRNA-133a family, weaken myocardial fibrosis, and reduce the damage caused by myocardial ischemia [65]. Therefore, Tongxinluo may play a regulatory role through the network of circNCX1/miRNA-133a/mRNA. Modulation of circHRCR/miR-223/ARC levels provides an attractive therapeutic target for the treatment of cardiac hypertrophy and heart failure [57]. TCM that tonifies *Qi* and activates blood (Shenmai injection, Shenqi Fuzheng injection, Danhong injection, and Qishen Yiqi pills) can significantly reduce the expression of miR-223-3p in the plasma of patients with AMI. Studies have also found that activating blood and dissolving phlegm can significantly reduce the expression level of miR-223 in the serum of hyperlipidemia rats in the myocardial ischemia-reperfusion model and play an anti-inflammatory role through the miR-223/PPRs pathway and inhibit apoptosis [66]. Ligustrazine, which is tetramethylpyrazine (TMP), can inhibit the proliferation, migration, apoptosis, and vascular remodeling of smooth muscle cells by downregulating the gene expression of miR-223 and then inhibiting the expression of CaMKIIδ [67]. Ginsenoside Rg1 upregulates the expression of eNOS by targeting miR-214, which is beneficial for promoting angiogenesis [68]. Electroacupuncture preconditioning can upregulate the expression of miR-214 and inhibit the increase in Ca^2+^, sodium/calcium exchanger 1 (NCX1), BCL2-like 11 (BIM), calmodulin-dependent protein kinase II*δ* (CaMKII*δ*), cyclophilin D (CypD), and other related proteins that have accumulated in hypoxia injury in H9c2 myocardial cells, and play a protective role in ischemia/reperfusion (I/R) [69]. The Fuzheng Huayu capsule has the effect of alleviating myocardial fibrosis after MI in rats. This mechanism may be related to upregulation of the expression levels in the miRNA-29 family and direct or indirect regulation of the ratio of matrix metalloproteinase 2/tissue inhibitor of metalloproteinases 2 (MMP2/TIMP2) and MMP9/TIMP1, improving the metabolic balance in the extracellular matrix [70].

## 6. Conclusion

Currently, the main method used to treat CHD in China is to diagnose and treat the disease using modern medicine to determine syndrome differentiation and combine that with the usage of TCM [71]. Numerous studies have confirmed the role of circRNAs and miRNAs in the regulation of the formation and development of AS. CircRNA and miRNA play important roles not only in the regulation of the vascular structure and function but also in the process of CHD and AS. As previously mentioned, circRNA targets are circANRIL (PES1), CDR1as (PARP, SP1), circRNA-HRCR (ARC), MFACR (MTP18), circRNA_010567 (TGF-*β*1), circNCX1 (CDIP1), circ-SATB2 (STIM1), circCHFR (FOXO1/CyclinD1), circNfix (Gsk3*β*), and circHIPK3 (a-SMA, COL1A1, COL3A1). Substantial research has been performed showing that miRNAs coordinate their mutual interaction between chronic endothelial repair and endothelial senescence and mechanistically link the regulation of macrophage cholesterol homeostasis with defective efferocytosis. MiRNA and its target gene (s) are let-7a-5p and let-7b-5p (LOX-1), let-7g-5p (LOX-1, TGFBR1 and SMAD2), miR-26a-5p (TRPC6), miR-126-3p (RGS16), miR-126-5p (DLK1), miR-217-5p (SIRT1), miR-216a-5p (BECN1), miR-34a-5p (SIRT1), miR-181b-5p (IPOA3), miR-92a-3p (KLF2, KLF4, SOCS5, and SIRT1), and miR-712-5p (Timp3) in the development of AS.

Single herb medicine, effective components, formulae, and Chinese patent medicines are widely used in the prevention and treatment of CHD. The functions of TCM in clinical treatment mainly include tonifying and regulating *Qi*, activating blood circulation, reducing phlegm, and relieving pain and consumptive disease. The treatment principles of PBSS in CHD are activating blood circulation and reducing phlegm, which are potential regulators of lipid metabolism and antiplatelet aggregation. However, due to the complex chemical components in CHM, its efficacy is actually a combination of various factors. Therefore, the biggest challenge for the modernization of TCM is to reduce the ambiguity of effective substances and mechanisms.

The circRNA-miRNA-mRNA network may be a new regulatory mechanism. CircRNAs and miRNAs interact with each other to regulate the mRNA expression of some critical genes in CHD genesis and progression. Previous studies showed that TCM contains active components that protect cardiomyocytes, act against atherosclerosis, inhibit apoptosis, promote cell growth, and protect the cardiovascular system by regulating miRNAs. Because there is a very tight connection among circRNAs, miRNAs, and mRNAs, it is likely that TCM may play a positive role in the treatment of CHD through the circRNA-miRNA-mRNA network. In conclusion, the circRNA-miRNA-mRNA network may be an important mechanism that can be affected by Chinese medicine, resulting in the amelioration of CHD. TCM can also be used to affect the circRNA-miRNA-mRNA network to assist with the diagnosis of CHD and the evaluation of the clinical effect of Chinese medicine on CHD.

## Figures and Tables

**Table 1:** Chinese clinical medicine for *XiongBi*.

Syndromes	Therapeutic principle	Formulae	Chinese medicines of formulas
Xin Xue Yu Zu	Huo Xue Hua Yu, Tong Mai Zhi Tong	Xuefu-Zhuyu decoction	*Taoren (Prunus persica (L.) Batsch), Honghua (Carthamus tinctorius L.), Danggui (Angelica sinensis (Oliv.) Diels), ShengDihuang (Rehmannia glutinosa Libosch.), Niuxi (Achyranthes bidentata Bl.), Chuanxiong (Ligusticum chuanxiong Hort.), Jiegeng (Platycodon、grandiflorum (Jacq.) A.DC.), Chishao (Paeonia lactiflora PalL), Zhike (Citrus aurantium L.), Gancao (Glycyrrhiza uralensis Fisch.), Chaihu (Bupleurum chinense)*
Qi Zhi Xin Xiong	Shu Gan Li Qi, Huo XueTong Luo	Chaihu-Shu gan San	*Chenpi (Citrus reticulata), Chaihu (Bupleurum chinense), Chuanxiong (Ligusticum chuanxiong Hort.), Xiangfu (Cyperus rotundus), Zhike (Citrus aurantium L.), Shaoyao (Paeonia lactiflora Pall), Gancao (Glycyrrhiza uralensis Fisch.)*
Tan Zhuo Bi Zu	Tong Yang Xie Zuo, Huo Tan Xuan Bi	(1) Gualou-Xiebai-Banxia decoction; (2) Di-Tan decoction	(1) *Gualou (Trichosanthes kirilowii Maxim.), Xiebai (Allium macrostemon Bge.), Jiangbanxia (Rhizome Pinelliae Preparata), Huangju (rice wine);* (2) *Fuling (Poria cocos (Schw.) Wolf), Renshen (Panaxginseng C.A.Mey.), Gancao (Glycyrrhiza uralensis Fisch.),Chenpi (Citrus reticulata Blanco), Danxing (Arisaema Cum Bile), JiangBanxia (Rhizome Pinelliae Preparata), Zhuru (Caulis Bambusae in Taenia), Zhishi (Citrus aurantium L.), Shichangpu (Acorus tatarinowii Schott)*
Han Ning Xin Mai	Xin Wen San Han, Xuan Tong Xin Yang	(1) Zhishi-Xiebai-Guizhi decoction (2) Si-Ning decoction	(1) *Zhishi (Citrus aurantium L.), Houpu (Magnolia officinalis Rehd. et Wils.), Xiebai (Allium macrostemon Bge.), Guizhi (Cinnamomum cassia Presl), Gualou (Trichosanthes kirilowii Maxim.);* (2) *Danggui* *(Angelica sinensis (Oliv.) Diels), Guizhi (Cinnamomum cassia Presl), Shaoyao(Paeonia lactiflora Pall), Xixin (Asarum heterotropoides), Tongcao* *(Tetrapanax papyrifer (Hook.) K. Koch), Gancao* *(Glycyrrhiza uralensis Fisch.), Dazao* *(Ziziphus jujuba Mill.)*
Qi Yin Liang Xu	Yi Qi Yang Yin, Huo Xue Tong Mai	(1) Shengmai San, (2) Renshen -Yang-Rongdecoction	(1) *Renshen (Panax ginseng C.A.Mey.), Maimendong (Ophiopogon japonicus), Wuweizi (Schisandra chinensis (Turcz.) Baill.);* (2) *Renshen (Panax ginsengC.A.Mey.), Baizhu (Atractylodes macrocephala Koidz.Atractylodes macrocephala), Fuling (Koidz.Atractylodes macrocephala Koidz.), Gancao (Glycyrrhiza uralensis Fisch.), Chenpi (Citrus reticulata), Huangqi (Astragalus membranaceus (Fisch.) Bge.), Danggui (Angelica sinensis (Oliv.) Diels), Baishao (Paeonia lactiflora Pall.), ShuDihuang (Rehmannia glutinosa), Wuweizi (Schisandra chinensis (Turcz.) Baill.), Guixin (Cinnamomum cassia Presl), Zhiyuan (Polygala tenuifolia Willd.)*
Xin Shen Yin Xu	Zi Yin Qing Huo, Yang Xin He Luo	1. Tian-Wang-Bu-Xin Dan, 2. Zhi-Gancaodecoction	(1) *Renshen (Panax ginseng C.A.Mey.), Fuling (Poria cocos (Schw.) Wolf), Xuanshen (Scrophularia ningpoensis Hemsl.), Danshen (Salvia miltiorrhiza Bge.), Jiegeng (Platycodon grandiflorum (Jacq.) A.DC.), Yuanzhi (Polygala tenuifolia Willd.), Danggui (Angelica sinensis (Oliv.) Diels), Wuweizi (Schisandra chinensis (Turcz.) Baill.), Maimendong (Ophiopogon japonicus), Tianmendong (Asparagus cochinchinensis), Baiziren (Platycladus orientalis (L.) Franco), Suanzaoren (Semen Ziziphi Spinosae), ShengDihuang (Rehmannia glutinosa Libosch.);* (2) *Gancao (Glycyrrhiza uralensis Fisch.), Shengjiang (Zingiber officinale Rosc.), Guizhi (Cinnamomum cassia Presl), Renshen (Panax ginseng C.A.Mey.), ShengDihuang (Rehmannia glutinosa Libosch.), Ejiao (Asini Corii colla), Maimendong (Ophiopogon japonicus), Maren (Cannabis sativa L.), Dazao (Ziziphus jujuba Mill.)*
Xin Shen Yang Xu	Wen Bu Yang Qi, Zhen Fen Xin Yang	(1) Shen-Fu decoction, (2) You-Gui Yin	(1) *Renshen (Panax ginseng C.A.Mey.), Fuzi (Aconitum carmichaelii Debx.);* (2) *ShuDihuang (Rehmannia glutinosa), Shanyao (Dioscotea opposita Thunb), Shanzhuyu (Cornus officinalis Sieb.et Zucc.), Gouqizi (Lycium barbarum L.), Gancao (Glycyrrhiza uralensis Fisch.), Duzhong (Eucommia u1moides Oliv.), Rougui (Cinnamomum cassia Presl), ZhiFuzi (Aconitum carmichaelii Debx.)*

**Table 2:** The pharmacological mechanisms of CHM.

VSMCs	Endothelial cells	Cardiomyocytes
Inhibiting expression or activity of contractile and structural proteins	Activation of NO signaling pathway	Alleviation of cardiac hypertrophy
Regulating expression of extracellular matrix proteins	Inhibition of inflammation	Attenuation of oxidative stress
Regulating calcium levels in a PKA/PKG/PKC-dependent way	Attenuation of oxidative stress	Inhibition of apoptosis
Attenuating proliferation and migration of VSMCs	Inhibition of apoptosis,	Opening K_ATP_ channels
Anti-inflammation improving mitochondrial function	Inducing angiogenesis suppression of endothelial permeability	ANP secretion

**Table 3:** The influence of active ingredients-miRNA in TCM on cardiovascular diseases.

Active components	Cell types	MiRNAs	Targets	Effects (references)
Tanshinone IIA	Macrophages	miR-375	KLF4	Anti-AS [33]
	NRCMs	miR-133	MAPK ERK1/2	Protecting cardiomyocytes [34]
	H9c2	miR-152-3p	PTEN	Inhibiting apoptosis [35]
Salvianolate	H9c2	miR-122-5p	Bax, Bcl-2	Inhibiting apoptosis [36]
Astragaloside IV	H9c2	miR-23a/miR-92a.	PI3K/AKT	Protects rat cardiomyocytes [37]
Notoginsenoside R1	H9c2	miR-21	PI3K/AKT	Cardioprotective actions [38]
Ginsenoside Rb1	NRCMs	miR-208	—	Protecting H/I impaired NRCMs [39]
Ginkgolide B	EPC	miR-126	Akt	Promoted cell growth [40]
Crataegus special extract	HUVEC	miR-155	eNOS	Protecting vascular [41]

EPC: endothelial progenitor cells; HUVEC: human umbilical vein endothelial cells; NRCMs: neonatal rat cardiomyocytes.

**Table 4:** The influence of gormulas of TCM-miRNA on cardiovascular diseases.

TCM formulas	miRNAs	Targets	Effects (references)
Compound Danshen dropping pills	miR-200b	VEGF/TGF-*β*1	Diabetic cardiomyopathy [42]
Fuzheng Huayu Capsule	miR-29	—	Myocardial fibrosis [43]
Xinfukang pill	miR-21	Fas/FasL	Inhibiting apoptosis [44]
Xinfukang pills	miR-1	Fibullin-2	Cardiac hypertrophy [45]
Qi li qiangxin capsule	niR-199a-5p	—	Cardiac remodeling and hypertrophy [46]

**Table 5:** The influence of PES1 (P) on cardiovascular diseases.

circRNA	miRNA (P/N)	MRNA (P/N)	Effect
circANRIL	—	PES1 (P)	AS [55]
CDR1as	miR-7a (P)	PARP, SP1 (P)	Myocardial infarction [56]
circRNA-HRCR	miR-223 (N)	ARC (P)	Myocardial hypertrophy [57]
MFACR	miR-625-3p (N)	MTP18 (N)	Apoptosis [58]
circRNA_010567	miR-141 (N)	TGF-*β*1 (P)	Myocardial fibrosis [25]
circNCX1	miR-133a-3p (N)	CDIP1 (P)	Apoptosis [59]
Circ-SATB2	miR-939 (N)	STIM1 (P)	Cell proliferation and differentiation [60]
circCHFR	miR-370 (N)	FOXO1/Cyclin D1 (N)	Migration of vascular smooth muscle [61]
circSlc8a1	miR-133a (N)	SRF, CTGF, Adrb1, Adcy6 (P)	Myocardial hypertrophy [62]
circNfix	miR-214 (N)	Gsk3*β* (P)	Cardiac regeneration [63]
circHIPK3	miR-29b-3p (N)	a-SMA, COL1A1, COL3A1 (N)	Cardiac fibroblasts [64]

P/N: positive regulation or negative regulation.
